# Mining the Microbiome and Microbiota-Derived Molecules in Inflammatory Bowel Disease

**DOI:** 10.3390/ijms222011243

**Published:** 2021-10-18

**Authors:** Matthijs Bekkers, Bojan Stojkovic, Gerard E. Kaiko

**Affiliations:** 1 School of Biomedical Sciences and Pharmacy, College of Health, Medicine and Wellbeing, University of Newcastle, Callaghan, NSW 2308, Australia; matthijs.bekkers@uon.edu.au (M.B.); bojan.stojkovic@newcastle.edu.au (B.S.); 2 Hunter Medical Research Institute, Newcastle, NSW 2305, Australia

**Keywords:** microbiome, inflammatory bowel disease, ulcerative colitis, Crohn’s disease, metabolomics, metagenomics, metatranscriptomics, microbiome therapeutics, clinical trials

## Abstract

The intestinal microbiota is a complex community that consists of an ecosystem with a dynamic interplay between bacteria, fungi, archaea, and viruses. Recent advances in model systems have revealed that the gut microbiome is critical for maintaining homeostasis through metabolic digestive function, immune regulation, and intestinal barrier integrity. Taxonomic shifts in the intestinal microbiota are strongly correlated with a multitude of human diseases, including inflammatory bowel disease (IBD). However, many of these studies have been descriptive, and thus the understanding of the cause and effect relationship often remains unclear. Using non-human experimental model systems such as gnotobiotic mice, probiotic mono-colonization, or prebiotic supplementation, researchers have defined numerous species-level functions of the intestinal microbiota that have produced therapeutic candidates for IBD. Despite these advances, the molecular mechanisms responsible for the function of much of the microbiota and the interplay with host cellular processes remain areas of tremendous research potential. In particular, future research will need to unlock the functional molecular units of the microbiota in order to utilize this untapped resource of bioactive molecules for therapy. This review will highlight the advances and remaining challenges of microbiota-based functional studies and therapeutic discovery, specifically in IBD. One of the limiting factors for reviewing this topic is the nascent development of this area with information on some drug candidates still under early commercial development. We will also highlight the current and evolving strategies, including in the biotech industry, used for the discovery of microbiota-derived bioactive molecules in health and disease.

## 1. Introduction

Most externally exposed mucosal surfaces and cavities in the human body are characterized by the presence of microbial communities composed of bacteria, archaea, viruses, and fungi [1]. The gastrointestinal tract is the location where the majority of human microorganisms reside, making up the gut microbiota, represented by several trillion microbial cells. This complex group of microorganisms is established shortly after birth and subsequently shaped by diet, geography, genetics, medications, and other lifestyle factors. Under homeostatic conditions, the gut microbiota is in a mutualistic relationship with the host, playing an important role in maintaining health through immune training, food metabolism, production of important metabolites, neuro-endocrine regulation, and protection from pathogens. However, disruption in the gut microbiota composition, also known as gut dysbiosis, has been linked with a number of chronic conditions ranging from metabolic disorders, such as obesity and type 2 diabetes, to immune-related diseases such as inflammatory bowel disease (IBD), arthritis, celiac disease, irritable bowel syndrome, food allergy, and asthma. In this narrative review, we will focus our discussion on IBD as it is furthest along the development pipeline for microbiota-based therapies.

IBD is a chronic disorder characterized by remitting and relapsing inflammation of the gastrointestinal tissue. IBD is classified into two subtypes: ulcerative colitis (UC) and Crohn’s disease (CD). UC normally affects the mucosal lining of the colon and rectum, while CD can affect any segment of the intestine and is normally transmural [2,3]. The disease etiology is unknown with multiple risk factors identified, including genetic susceptibility, environmental factors, the intestinal microbiota, and the interplay between all three. The aim of current treatment is to induce and maintain remission, prolong the low activity disease course, and prevent complications. Treatment options are mainly limited to immunosuppressant and biologic therapy [2,3]. However, more than half of the patients require surgery during their lifetime, and mucosal healing and long-term remission are often hard to achieve. Therefore, there is a strong clinical need to investigate other avenues for improved treatment of IBD. The interrogation of the functional potential of the human gut microbiota offers a unique opportunity for discovery of new therapeutic candidates for IBD. As technologies have advanced, an increasingly reductionist discovery approach is possible for microbiota-derived therapies, and this includes: fecal microbiota transplantation, individual species or consortia as live biotherapeutic products or probiotics, bioactive metabolite identification, and large-scale sequencing studies to identify gene/protein molecules with therapeutic potential. We will review each of these approaches here.

## 2. Taxonomic Characterization of the Gut Microbiome in IBD

The Human Microbiome Project (HMP) was among the first comprehensive large-scale characterizations of the healthy gut microbiome [4]. The HMP demonstrated that the intestinal microbial diversity is dominated by two large phyla: *Bacteroidetes* and *Firmicutes*, while *Proteobacteria* and *Actinobacteria* are also commonly present but in a significantly lower abundance.

A number of studies have investigated the intestinal microbiota dysbiosis that occurs in IBD. The main hallmark of the gut microbiome change that occurs is the overall loss of diversity and reduction in obligate anaerobic bacteria [5,6]. Early cross-sectional studies based on 16S profiling identified a depletion in commensal bacteria reflected by a decrease in *Firmicutes* and *Bacteroidetes* and increase in *Gammaproteobacteria*, primarily *Escherichia* [5,7,8]. In an attempt to address some of the inconsistencies in the dysbiotic profiles reported in earlier studies and to identify issues with the cause and effect relationship between microbiome changes and disease, Morgan et al. [9] published one of the first studies to highlight the confounding factors in microbiome analysis such as age, lifestyle, medication, and the intestinal region affected. The authors reported depletion in *Roseburia* and *Phascolarctobacterium*, and an increase in *Clostridium* and *Escherichia*. Importantly, they demonstrated that the shifts in metabolic pathways were more dramatic than underlying taxonomic changes in the gut microbiome. Gevers et al. [10] performed one of the first studies of IBD dysbiosis using a large cohort of newly diagnosed and treatment-naive pediatric patients to eliminate the confounding effect of medication on the microbiome. They confirmed the results from Morgan et al. but also identified an increased abundance of *Pasteurellaceae* (*Haemophilus* sp.), *Veillonellaceae*, *Neisseriaceae*, and *Fusobacteriaceae*, and a reduction in *Bacteroides*, *Faecalibacterium*, *Roseburia*, *Blautia*, *Ruminococcus*, and *Coprococcus*, in patients with CD [10].

Despite the fact that a significant number of studies have reported an association between gut microbiota dysbiosis and chronic disease, demonstrating the cause and effect relationship remains difficult. The description of taxonomic changes using 16S sequencing is mostly limited to higher phylogenetic groups, at the genus/family level, obscuring some of the specificity of the changes. The emergence of longitudinal studies and the shift from 16S sequencing to a whole metagenome shotgun approach has enabled better resolution by identifying species and even strains associated with IBD. Metagenomic sequencing also enables identification of specific microbial genes and pathways, which allows important functional characterization.

Minimizing confounders through precise patient grouping and selection as well as shotgun sequencing introduces greater precision into microbiome studies, but another powerful tool is the use of longitudinal patient studies. Schirmer et al. [11] studied a large longitudinal IBD cohort, where patients were followed for one year with biweekly stool samples for paired metagenomic and metatranscriptomic analysis. The results demonstrated that changes in the taxonomic composition over time often correlate with shifts in disease severity during the course of IBD disease. Given the relapsing and remitting nature of IBD as well as the variability of the microbiome, this reinforces the importance of longitudinal microbiome studies. A further study on the same cohort by Lloyd-Price et al. [12] demonstrated that IBD is accompanied by a reduction in *Faecalibacterium prausnitzii* and *Roseburia hominis*, while *Escherichia coli* and *Ruminococcus gnavus* are enriched. This study also confirmed the importance of including information on the disease activity, and fluctuations that occur in the same patient over time when characterizing the dynamics of dysbiosis in IBD. The authors demonstrated that temporal taxonomic changes are significantly more pronounced in patients with IBD as compared to non-IBD control subjects [12]. Both of these studies clearly demonstrate the importance of a multi-omic longitudinal approach in better understanding IBD onset and progression. By integrating both genomic and transcriptomic information, we are able to complement the taxonomic changes with the functional potential of specific microbial species.

**Figure 1:**
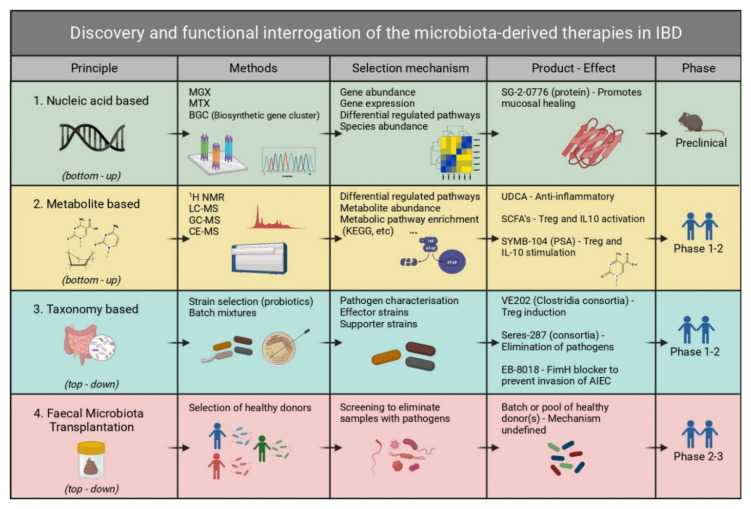
Discovery and functional interrogation of microbiota-derived drug candidates in IBD. Methods for discovery of microbe-derived drugs for IBD can broadly be divided into bottom-up (sequence or metabolite based) and top-down (taxonomy or FMT based). A bottom-up approach refers to starting with the basic building blocks or individual components of the microbiome, i.e., metabolites or genes, working your way up through screening these many components, and identifying their function. A top-down approach starts with a complex mixture, i.e., the whole microbiota or part thereof, followed by narrowing down functional components by positive selection on sub-fractions of the pool. For nucleic acid-based methods, the MGX (metagenomics) and MTX (metatranscriptomics) technologies provide information about the relative abundance of species, genes, and transcripts. Metabolite-based methods apply analytical techniques such as MS to discover metabolites that are differently abundant in IBD. Taxonomy-based studies use a gnotobiotic approach that starts from complex microbiota samples and narrows these down to effector strains, after which their mechanism of action is investigated. In addition, drugs that are designed to reverse dysbiosis also fall in this category. Therapies based on FMT aim to identify donors with microbial communities that induce remission upon transplantation, without delineating specific mechanisms or factors. Whereas most nucleic acid- and metabolite-based drugs are in the preclinical phase, several taxonomy- and FMT-based drugs have passed the first clinical trial stage. FMT, fecal microbiota transplantation; MGX, metagenomics; MTX, metatranscriptomics; MS, mass spectrometry; LC-MS, liquid chromatography-mass spectrometry; GC-MS, gas chromatography-mass spectrometry; CE-MS, capillary electrophoresis-mass spectrometry; KEGG, Kyoto Encyclopedia of Genes and Genomes; PSA, polysaccharide A; UDCA, ursodeoxycholic acid. Included in the product effect column are several examples of microbe-derived drugs for IBD in a preclinical or clinical trial phase. Created with BioRender.com.

Within the *Escherichia coli* species, a specific pathogenic strain better known as adherent-invasive *E. coli* (AIEC) has been found to be highly prevalent in ileal Crohn’s disease (ICD) [13]. The identification of taxonomic shifts associated with IBD by researchers has led to the development of several therapeutic strategies to restore homeostasis. One example of these strategies is being investigated by Enterome, which has developed a small molecule FimH blocker (Figure 1, Table 1) that prevents AIEC from adhering to and subsequently invading the intestinal epithelium [14].

## 3. Fecal Microbiota Transplantation

Fecal microbiota transplantation (FMT) is a method of introducing ‘normal’ donor feces from healthy individuals into disease-affected patients. This method is already used in the clinic with a >90% success rate as a last resort therapy for antibiotic-resistant *Clostridioides difficile* infection [15,16]. There are now a multitude of other disease conditions undergoing Phase 2/3 trials with FMT. However, despite its use in the clinic for over a decade, multiple serious adverse events have been reported recently, which reveals our limited understanding of this treatment, and its lack of control as an appropriate or safe long-term therapy [17]. Early studies have pointed out that FMT shows efficacy in patients with UC [18]. This has been explored by double-blind, randomized controlled trials (RCT) on UC that measured clinical or endoscopic remission upon FMT treatment [19,20,21,22]. Although there is no formal definition of remission, this typically includes a certain score improvement in the clinical assessments such as the Mayo endoscopic score or the full Mayo score [23]. A systematic meta-analysis review that compared four RCTs on patients with UC found an overall clinical or endoscopic remission rate after 8 weeks of 37% (n = 64/278) in patients receiving FMT compared with 18% in those receiving placebo [24]. This compares favorably with biologic therapies targeting TNF-α, IL-23, and α₄β₇ integrins already approved in the clinic.

Despite its efficacy, FMT has not received clinical approval for UC and CD treatment in any country and is currently still in a trial phase due to major issues with regulatory approval in this area of ‘live therapeutics’. There remain other major hurdles for FMT outside of its use in *C. difficile*. It is unclear how many doses of FMT need to be administered for chronic diseases such as UC, whether it should be administered via enema or orally, whether pre-treatment with broad-spectrum antibiotics is important to create a niche, and, most importantly, where the donor material should be sourced from. There are major issues as well with how accurately it can be screened given the amount of unknowns in the microbiome—one can only run targeted screens for what is known. From the RCT studies, there is clearly a therapeutic signal for IBD, and it can be concluded that FMT likely contains therapeutically important bioactive molecules. However, these therapeutic bioactive molecules or microbe strains remain largely unknown. Although remission numbers look promising, FMT’s nature as a ‘black box’ therapy, inability to be standardized, and risk of serious side effects suggests it is not a long-term solution for the treatment of IBD. From a manufacturing and wide-scale adoption perspective, FMT is not really feasible considering the nature of the sample, inconsistency between batches and between individual donors, lack of quality control, and storage variability. A superior alternative is to identify the therapeutically important bioactive molecules and microbial strains in the FMT mixture as candidates for treatment.

## 4. Live Biotherapeutic Products

There is some evidence to suggest that the effects of FMT work beyond the transient transplantation of bioactive effector molecules from donor to recipient in the FMT mixture given the long-term remission in certain diseases. This suggests that the introduction of specific donor strains may play an important role in reducing disease activity in FMT [19]. Host–microbe interactions along the GI tract that exhibit immunomodulatory effects on the host have been extensively studied [25,26,27]. One common approach to investigate these interactions is by colonizing a gnotobiotic mouse model with wildtype microbiota species and/or engineered bacterial strains [28]. One of the first real demonstrations of this approach arose from the seminal work by Sarkis Mazmanian and colleagues with the identification of polysaccharide A (PSA), produced by the symbiont *Bacteroides fragilis*, which protects against *Helicobacter hepaticus*-induced colitis [29]. In animals harboring *B. fragilis* not expressing PSA, *H. hepaticus* induces colitic disease and pro-inflammatory cytokine production in the colon [29]. In addition to *B. fragilis*, other gut-resident bacteria such as Clostridiales have been demonstrated to trigger regulatory T cells, or signaling pathways such as the activation of the colitogenic Th1 and Th17 responses by AIEC, segmented filamentous bacteria, and *Citrobacter rodentium* [30,31,32]. Consequently, the use of live biotherapeutic products that either add to anti-inflammatory mechanisms or counteract these pro-inflammatory mechanisms might be a strategy to restore homeostasis and immune tolerance in the gut.

The term live biotherapeutic product (LBP) has been defined as live organisms designed and developed to treat, cure, or prevent a disease or condition in humans [33]. Probiotics’ and LBPs’ main point of difference is their labeling with regard to regulatory claims; however, some probiotics could be classified as LBPs upon RCT completion and passing specific criteria [34]. LBPs can be administered as dormant spores, micro-encapsulations, or freeze-dried whole bacteria and are intended to permanently reside in the gut when used for treatment [33]. The advantages of LBPs over purified molecules include the ability to continuously deliver effector molecules on site in specific niches in the gut in which they reside, avoiding administration of high doses of bioactives [35]. However, issues remain over competitive fitness with the endogenous microbiota, and safety concerns about administering live microorganisms. To date, there are no approved LBPs for use in IBD, and clinical trials remain ongoing.

Although originally not classified as an LBP, one of the first live probiotic strains used in the treatment of IBD was EcN 1917 (*Escherichia coli* Nissle 1917). This non-pathogenic Gram-negative strain is a well-known probiotic that expresses factors such as microcins, adhesins, and proteases that presumably support its survival and colonization of the human gut that typically occurs within several days [36]. Currently, lyophilized *E. coli Nissle* is available as Mutaflor^®^ and is the only probiotic recommended by European Crohn’s and Colitis Organisation guidelines as an effective alternative to mesalazine in the maintenance of remission in patients with UC [37]. Mutaflor^®^ showed a similar efficacy (61.6%) for UC remission when compared to mesalazine (69.5%) based on a meta-analysis conducted in 2015 including six trials [38]. Countries where Mutaflor^®^ is registered and available as a probiotic drug include Germany, Canada, Singapore, Australia, and New Zealand. Its potential as an LBP for wider IBD treatment is being further explored [37,38], and genetic engineering preclinical studies of EcN are ongoing to create a more effective LBP [39].

Many potential LBPs for treatment of IBD were explored in double-blind RCTs almost two decades ago. Among these candidates were single-strain therapies such as *Lactobacillus GG* [40] or multi-strain cocktails also containing *Bifidobacteria* [41,42], all of which have shown inconsistent results. A 2010 meta-analysis concluded that probiotic treatment of this type with ‘digesta or flow-through bacteria’ was more effective than placebo in maintaining remission in UC; however, the results were not better than those of standard treatments such as 5-ASA or mesalazine [43]. Overall, these types of older-style probiotics do not show promising results to treat or maintain remission in IBD, and therefore the wider exploration of LBPs, especially anaerobic strains, has continued.

More recent trials of LBPs in IBD include a consortium of 17 *Clostridia* strains developed by Vedanta Biosciences, currently in Phase 2 clinical trials for UC and CD treatment (Figure 1, Table 1). The strains demonstrate evidence of induction of Treg cell expansion in the gut mucosa [44,45]. This consortium mixture was discovered using a top-down gnotobiotic approach by transferring human-derived fecal microbiota after chloroform treatment (removes all but spores) into germ-free mice, after which subsequent mouse-to-mouse transfers were carried out while selecting for the specific anti-inflammatory Treg expansion [44]. In addition to Vedanta, Seres Therapeutics is another company that is focused on the development of LBPs for IBD treatment with SER-287, which is a consortium of multiple *Firmicutes* spores and is currently in a Phase 2 clinical study (Figure 1, Table 1) [46]. A more recent LBP from Seres Therapeutics that has entered Phase 1 trials is SER-301, which comprises a set of 18 live human commensal bacterial strains that are purified and cultured from the stool of healthy donors. In addition to these consortium mixtures of strains, the use of single-strain LBP *Bacteroides thetaiotaomicron* (Thetanix) has demonstrated enhanced mucus production in the colon of gnotobiotic mouse models and is being developed by 4D Pharma for treatment of IBD (Table 1) [47]. The origin of strains used in these ‘live pharmaceuticals’ is expected to be of major importance when it comes to proceeding to clinical studies and regulatory approval of LPBs. The legislation on this issue has been subject to change in recent years and is an evolving area [48]. Strains isolated from stool will likely be assessed differently from genetically engineered strains by authorities, and at present, the FDA requires additional testing to guarantee the stability of genetic modifications [49]. Genetically engineered strains are likely to face serious regulatory issues in Europe and other jurisdictions where existing bans on the use of GMOs will pose a major hurdle. Bacterial engraftment is essential in treatments with LPBs, and although many efforts are ongoing to increase the understanding of the dynamics of strain engraftment, the limited ability to predict bacterial engraftment in different individuals with different microbiomes remains one of the major hurdles in the transition of LBPs into the later stages of Phase 2/3 clinical trials [50].

Although not classified as LBPs, bacteriophages that are known to target well-defined species of bacteria are currently being explored as therapies to modulate bacteria linked to IBD. For example, AIECs that are highly prevalent in ICD [13] have been shown to be specifically depleted by bacteriophage therapy in preclinical studies [51]. Ecoactive, a bacteriophage developed by Intralytics, targets AIECs and is currently in the recruitment stage of a Phase 1/2a study to assess the safety and efficacy of its treatment of patients with CD (Table 1). Another bacteriophage cocktail for IBD treatment has been developed by BiomX. Their candidate BX003 targets *Klebsiella pneumoniae*, which is a Gram-negative opportunist pathogen associated with the onset and exacerbation of primary sclerosing cholangitis and IBD (Table 1) [52]. Bacteriophages hold self-replicating features and are thought to be inert to mammalian cells. They are increasingly thought of as potent antimicrobial agents for therapies to treat IBD; however, the ‘live therapy’ nature of these products represents a major regulatory challenge to overcome. Bacteriophage-based therapeutics may have more targeted effects than antibiotics, which are still used rather non-specifically in many clinical settings to treat IBD, and they may overcome some of the potential engraftment issues associated with LBPs.

## 5. Metabolomics of the Gut Microbiome in IBD

Metabolomics focuses on the investigation of small molecules (<1500 Da) in various types of biological samples such as tissues, serum, feces, or urine. Recent advances in analytical methods have accelerated discoveries in metabolomics. In a typical metabolomics study, a separation method is paired with spectroscopy-based analytical techniques, including nuclear magnetic resonance (NMR), gas chromatography mass spectrometry (GC-MS), or liquid chromatography mass spectrometry (LC-MS). NMR performs best in the analysis of targeted metabolites but suffers from poor sensitivity [53]. GC-MS analysis is a highly versatile analytical technique due to its robustness, good separation capacity, selectivity, sensitivity, and reproducibility [54]. This method is mainly used to characterize volatile compounds with a low molecular weight (50–600 Da) and high thermal stability. In order to analyze non-volatile agents, a chemical derivatization needs to be performed on the target metabolite. This means that specific measurements are conducted on the derivative rather than the target metabolite [54]. In contrast to GC-MS, LC-MS does not require metabolite derivatization and can analyze a wide range of metabolites [53]. No single analytical technique is suitable for all metabolites, and often a combination of different methods must be used when a broad characterization of the ‘global’ metabolome is required [53]. Metabolite identification can be targeted, untargeted, or a combination of the two [55]. Targeted metabolomics is based on pre-existing knowledge of the candidate molecule/s and reference standards (rational hypothesis studies), whereas untargeted metabolomics (discovery studies) aims to identify all metabolites in a given sample.

The gut microbiota is the source of a wide range of metabolites which are produced by bacteria and fungi directly or through the modification of dietary and host molecules [55]. Microbiome-derived metabolites are strong candidates for a causal link between gut dysbiosis and IBD, due to their diverse functional effects on the host, abundant production, ability to cross both the mucus and intestinal epithelial barrier, and chemical stability in the gut. Targeted studies have identified a number of metabolites associated with IBD. Short-chain fatty acids (SCFA) are important microbial by-products of fiber fermentation in the gut, of which acetate, propionate, and butyrate are the three most abundant metabolites [55]. These compounds have anti-inflammatory effects through Tregs and IL-10 activation [56]. SCFAs also play a role in maintaining epithelial integrity through NLRP3 inflammasome-driven activation and IL18 induction [57]. Finally, butyrate is a major source of energy for colonocytes and regulates intestinal epithelial barrier repair [58]. Importantly, levels of SCFAs have been found to be decreased in the fecal samples of patients with IBD relative to non-IBD controls, suggesting they not only have a functional effect but are also dysregulated in disease [59,60]. Therefore, the use of SCFAs as supplements, in theory, would be a promising approach in the treatment of IBD [58]. However, the clinical trials on SCFAs in IBD to date have produced very mixed results. A very small early proof-of-principle study that examined the effect of butyrate enemas on 10 patients with UC demonstrated an overall improvement in the clinical illness, with a reduction in the endoscopic score and the histological degree of inflammation [61]. Breuer et al. [62] conducted a larger trial involving 103 patients with distal UC treated with enemas containing butyrate, acetate, and propionate. Unfortunately, the difference in the proportion of patients achieving clinical remission was insignificant. In addition, none of the histological changes following enemas reached statistical significance. A recent review by Jamka et al. [63] compiled all IBD clinical trials to date using butyrate enemas, including 8 studies and 227 patients with UC. They concluded that butyrate enema treatment in UC does not result in a significant improvement in disease activity index, endoscopic, or histological scores [63]. The prominent reason suggested to explain this result is that the short half-life of butyrate in vivo prevents its efficacy, and efforts to alter the chemical structure, micro-encapsulate it, or improve dietary production by fiber are ongoing. Two clinical trials assessing the ability of butyrate supplementation to improve clinical scores in IBD are currently ongoing (Table 1).

Bile acids are another class of small molecules that have critical metabolic and immune effects in the gut and are dysregulated in IBD. Primary bile acids, produced in the liver, have an important role in lipid digestion and absorption. Secondary bile acids are produced by the intestinal microbiota through primary bile acid deconjugation [55]. Both primary and secondary bile acids can interact with a number of receptors, which leads to downstream immune effects, such as suppression of NF-kB, IL1, IL6, and TNFα [55]. In the small intestine, primary bile acids can be modified by highly conserved bacterial bile salt hydrolases to produce unconjugated bile acids [64]. Further bile acid modifications occur in the colon, giving rise to lithocholic and deoxycholic acids, as the most abundant secondary bile acids [65]. Sinha et al. [66] quantified the levels of lithocholic and deoxycholic acids in the stool of patients with UC. They found that the levels of these two compounds are reduced in patients with UC, which is accompanied by a lower abundance of genes and taxa associated with primary-to-secondary bile acid conversion. The authors also demonstrated that bile acid supplementation had a protective effect in a colitis mouse model. Ursodeoxycholic acid is another secondary bile acid that is significantly less abundant than lithocholic and deoxycholic acids but may have beneficial effects in the gut through barrier protection and its anti-inflammatory action [67]. This secondary bile acid is currently being investigated in an ongoing Phase 2 clinical trial for its ability to reduce inflammation in IBD (Table 1).

The majority of targeted metabolomics studies in IBD used adult patients undergoing treatment, which could mask the true correlation between IBD disease activity status and metabolite levels. To overcome this issue, Kolho et al. [68] quantified a number of targeted metabolites in a newly diagnosed treatment-naive pediatric IBD cohort. They reported altered metabolic pathways including amino acid and sphingolipid metabolism, the urea cycle, and bile acid, folate, and pterin biosynthesis.

In contrast to targeted methods, untargeted metabolomic studies offer an unbiased characterization of previously unknown metabolite–disease correlations [55]. In a large cross-sectional comparison of stool metabolic profiles between patients with and without IBD, Franzosa et al. [69] reported significant and diverse perturbations in the metabolite composition, where 31% of all metabolic features were differentially abundant among IBD patients versus patients without IBD. Of these, the vast majority (71%) were significantly reduced in IBD, matching the known loss of bacterial taxa diversity from sequencing studies (as described above). It is important to note that when conducting untargeted metabolomics studies, both microbiota and host/diet metabolites will be detected, and many metabolites can be derived from both sources. The enrichment analysis identified changes in broad metabolic classes such as increases in sphingolipids (host and microbiota derived) and bile acids (host and microbiota derived) and a reduction in triacylglycerols (diet) and tetrapyrroles (host and microbiota derived) in IBD. The authors also developed a model that utilizes metabolic profiles to classify samples according to their IBD status, with very high sensitivity. Lloyd-Price et al. [12] also performed a non-targeted longitudinal multi-omics analysis including metabolic profiles in a mixed pediatric and adult IBD cohort over the course of one year. They also demonstrated that metabolite diversity is lower in patients with IBD. The levels of vitamin B3 (host/diet and microbiota derived) and B5 (diet and microbiota derived) were particularly reduced in IBD. In contrast, nicotinuric acid was almost absent from healthy patients while highly abundant in IBD [12]. The same group followed up this large-scale metabolomics analysis with functional validation of a subgroup of metabolites [70]. They identified a number of compounds that modulate the growth of intestinal bacteria. Specifically, they showed that N-acetylcholamines, a class of signaling lipids, are elevated in the stool of patients with IBD and demonstrated in vitro that these molecules support the growth of the same bacteria known to be increased in IBD while inhibiting the growth of bacterial species depleted in IBD.

An alternative method for putative metabolite identification involves quantification of the DNA abundance of biosynthetic gene clusters (BGCs), which are large groups of closely linked genes encoding enzymes involved in the same metabolic pathway that produce small secondary metabolites as end products [71]. The identification of enrichment of a specific BGC can then facilitate further metabolite discovery. Due to their conserved nature, BGC homologues can be identified and quantified across different taxa. Furthermore, knowledge of a specific BGC can enable the production of a complex metabolite without the need to isolate the chemical or bacteria responsible for its production. Ongey et al. [72] employed molecular heterologous methods to isolate, clone, and overexpress in *E. coli* the BGC metabolic machinery needed for the production of ruminococcin A, a lanthipeptide with antimicrobial properties and a product of *Ruminococcus gnavus*, which is known to be altered in IBD stool. A reverse approach is also possible, where the BGC identification follows metabolite identification in order to further characterize the properties of the bioactive pathway. Henke et al. [73] successfully identified and isolated a pro-inflammatory polysaccharide, glucorhamnan, from a *R. gnavus* supernatant, followed by the characterization of a BGC responsible for glucorhamnan biosynthesis. They identified a gene within the cluster that is responsible for the glucorhamnan transfer to the bacterial cell wall. The treatment of the cell wall with a lysozyme-like enzyme enabled the release of glucorhamnan into the supernatant, improving the metabolite yield. Wlodarska et al. [74] found that the gut bacterial species *Peptostreptococcus russelli* has a protective effect in the DSS colitis mouse model through stimulation of goblet cell and mucin production to improve the intestinal barrier. To further dissect the mechanism of this effect, they investigated the complete genome of *Peptostreptococcus russelli* and identified the phenyllactate dehydratase BGC gene cluster (fldAIBC), which is involved in tryptophane metabolism. Further MS analysis of the *Peptostreptococcus russelli* supernatant detected two tryptophane metabolites: indole-3-propionic acid (IPA) and indoleacrylic acid (IA). In vitro functional analysis of the IPA and IA effects on bone marrow-derived macrophages and colonic spheroids identified elevated IL10 production, reduced TNFα production, and increased expression of genes involved in goblet cell function. The investigation of BGC-derived metabolites is only in its infancy in this field and will be an area of significant future opportunity for discovery.

## 6. Sequencing-Based Approaches

Nucleic acid sequencing approaches to discovery in the microbiome have exponentially expanded with the advent and cost reductions of next-generation sequencing technology. Due to its convenience, most sequence-based studies that investigated the activity and differential abundance of the microbial community in IBD focused on the collection of stool samples. Other methods including intestinal biopsy (99% eukaryotic cell reads) and mucosal swab/lavage sampling have shown some differences in the microbial composition with stool, which has been suggested to be driven by mucosal-associated microbes; however, these methods suffer from a low level of microbial reads, and thus the resolution of the data has its own limitations [75]. Furthermore, mucosal sampling assumes that intimate microbe proximity to the intestinal epithelium is key, but this ignores the importance of the secretion of diffusible bioactive molecules from the microbiota. Likely, both mechanisms are important for host–microbe interactions in the gut. Comparisons of stool sample storage methods using RNAlater or ≥95% ethanol concluded that the variation observed in metagenomic (MGX) studies can be primarily explained by inter-individual differences and substantially less so by the collection or storage method [76,77]. After sample collection, DNA/RNA is isolated using cell lysis. Determining the method of cell lysis requires careful consideration, since certain methods such as chemical lysis have been shown to have effects on DNA quality and thus introduce bias in the downstream sequencing process [78]. Mechanical lysis methods such as bead beating and heating (to lyse more difficult Gram-positive species) reduce bias from cell lysis and are typically regarded as the preferred option over protocols using solely chemical lysis [79]. There are different approaches to sequencing-based discovery methods including 16S rRNA and whole metagenome sequencing.

### 6.1. 16S rRNA Sequencing

16SrRNA sequencing is the most widely used method in the exploration of the taxonomy of microbial communities mainly because of the lower costs and well established methods of bioinformatics analysis by hierarchical taxonomic classification. This method is based on amplification and sequencing of the 16S hypervariable regions of DNA, and clustering of sequences based on similarity, in order to generate operational taxonomic units (OTUs), which allows for family/genus-level resolution of detection [79,80]. The main advantages of using 16S sequencing are the ability to avoid host contamination and the low relative cost due to the short read, and high-throughput nature of this method (Table 2). The technology enables research groups to answer questions about microbial taxa present in their samples and can even provide some level of modest prediction of the community’s functional potential. However, because of its inability to directly identify microbial genes, its limitations in taxonomic resolution, and the introduction of primer bias due to its use of PCR, the value of the exclusive use of 16S rRNA sequencing in the search for new microbiota-derived drugs is problematic (Table 2) [80,81]. In addition, it has been demonstrated by multiple studies that low-abundance bacteria in microbial communities are poorly detected by 16S sequencing, and techniques such as shotgun metagenomics are more suited for this purpose [82,83,84]. Although the bioinformatics tool PICRUSt2 can assist in deriving certain functional information from 16S sequencing, it relies on inferring functions based on genes known to exist in defined taxa [85]. This use of inference to derive an extra level of biological information from 16S data lacks the sensitivity to identify much of the actual microbial genome in the sample and also adds an inherent bias to the functional analysis because many different species and strains have different genes/functions despite registering the same 16S taxa profile. More recently, full-gene length 16S sequencing has enabled better resolution of bacterial detection at the species and strain levels and may alleviate some of the concerns about functional interrogation and specificity [86,87].

### 6.2. Whole Metagenome Shotgun Sequencing (Metagenomics) and Analysis

In contrast to 16S sequencing, shotgun metagenomics enables the interrogation of the relative abundances of microbes such as bacteria, fungi, and viruses in the sample and, perhaps more importantly, uncovers the relative abundance of microbial genes to begin functional interrogation. Besides this, its ability to distinguish between taxa at greater resolution enables researchers to identify bacteria at the species or, in some cases, even at the sub-species and strain level. Its most important power, however, is not the taxonomic resolution but instead its capacity to identify millions of microbial genes and hence the functional potential of a sample (Table 2). Results from the large-scale HMP consortium showed that across different body sites, the taxonomic composition of the microbiome is highly variable among individuals; however, the function of the microbial communities as defined by metabolic gene pathways was highly conserved [4]. This demonstrates that different genera and species can share many functions and pathways, which indicates that many genes in the microbiome are likely conserved among different community members with potentially some redundancy. Although metagenomic studies have the ability to detect microbial genes from any type of microbe in the intestinal tract, in reality, due to costs, limitations on sequencing read depth, and abundance in the gut, most metagenomic studies detect very little fungal or viral genomes (<1%) and are primarily composed of bacteria and archaea. Studies on fungal and viral genomes in the gut microbiome require a specific methodological design and enrichment, and in the case of fungi, they rely on sequencing of the ITS1 region. Studies on the relationship between IBD and viral or fungal species have revealed an increased abundance of *Candida* taxa [88] and *Caudovirales* [89] and a decrease in *Caudovirales* diversity in the mucosa [90]. However, despite these correlations, very little is known about how genes or biomolecule products of these microbes may contribute to IBD. This will require an enormous sequencing depth and advances in the annotation of fungal and viral genomes.

In many cases of metagenomic sequencing, there is contamination of samples with host-derived sequences, which can be partly resolved by prior depletion of mammalian genomic material through chemical lysis methods (Table 2) [91]; however, in the context of fecal stool sampling, where microbial DNA is typically more abundant than host-derived material (<1%), post-sequencing filtering of host-derived sequences by bioinformatics tools is a preferred option [69,92]. After the sequencing data have passed the quality control (QC) phase, the critical assembly process takes place, where the sequencing output is translated into information on function and taxonomy. Assembly attempts to arrange the reads together into larger contigs using complex algorithms. The choice of assembly algorithm remains critical for optimal downstream analysis since many considerations need to be made in this process that are dependent on the sample type, species complexity, sequence depth, read quality and length, availability of reference databases, and the purpose of the study [75,93]. Different bioinformatics tools are available to perform these steps, and their choice is dependent on the aforementioned considerations. For de novo assembly, as opposed to reference-based read mapping, the process requires a large computational capacity that limits the speed and efficiency of this step. For a typical metagenomics study, some of the most widely used tools for assembly are Megahit, metaSPAdes, RayMeta, SOAPdenovo2, and IDBA-UD [35,94,95,96,97]. These tools employ algorithms that are based on De Bruijn graphs, which have been shown to be of great value in arranging millions of reads by the use of k-*mers*, especially in the case of de novo assembly [98]. After arranging sequencing reads into larger contigs, contig binning is performed to construct a taxonomic profile by aligning contigs against reference datasets. More recently, novel methods have been developed that include supervised and unsupervised binning in order to build taxonomic profiles more effectively [93,99]. Accurate taxonomic profiling and microbial gene identification remain challenging and require a high sequencing depth and coverage, and thus a high processing capacity is needed, especially in situations where a community contains many closely related species such as the microbiome [100,101,102]. The accuracy of mapping the reads to the correct taxa is only as good as the prior known microbial genomes, and therefore access to up-to-date high-quality reference databases is at least as important as the quality of the classification tools. Reference-based metagenomics mapping is a common practice in human microbiome-based research and is highly effective, and it also avoids the highly demanding computational assembly processes involved in de novo assembly. However, despite ample work being carried out to fill the gaps in microbial genomes and identify and sequence previously unknown microbiota strains, there are still countless non-annotated reads in metagenomic studies [103]. Common classification tools used in microbiota reference-based studies are Kraken2 [104], MetaPhlAn2 [105], and Centrifuge [106] which use specific marker genes to identify taxa in the sequencing data.

One of the strengths of shotgun metagenomics is the ability to investigate the functional and metabolic roles of microbiome member species by utilizing tools that include approaches based on homology, motifs, or context [93]. As this method is based on DNA sequencing, it can only provide information on the functional potential. This is a limitation of metagenomics for which no straightforward solutions currently exist except for pursuing an approach that includes other omics technologies such as metatranscriptomics or metabolomics.

MGX studies typically use short read sequencing which limits the sequencing of short tandem repeats highly prevalent across genomes [107]. Newer formats for long read sequencing are able to sequence read lengths up to 200 Kbp and have demonstrated the ability to sequence complete bacterial genomes from stool samples. This technology could potentially overcome the limitations of the sequencing of short tandem repeats [100] and, in addition, could aid in completing bacterial genome assemblies [108]. An even more recent technology that may offer further insights beyond the bulk sequencing of MGX studies is single-cell metagenomic sequencing [109]. As is the case for mammalian cell studies, one of the key advantages over shotgun MGX technology is that it allows for sequencing of rare subpopulations of microbes that would be missed by the averaging effect and detection limits of bulk MGX sequencing. Through FACS or microfluidics, single bacterial cells are isolated and, after unique barcoding of their genomes, can be separately analyzed [110]. This area is still in its infancy and rapidly evolving, but it is likely to yield important data in the future on less abundant microbiota bacteria that reside in specific niches perhaps in the IBD mucosa.

One of the first studies that used shotgun MGX to interrogate differences in the microbiome gene units rather than just taxa between healthy individuals and patients with IBD was conducted by Morgan et al. [9]. The authors found changes in oxidative stress pathways, decreased carbohydrate metabolism, and demonstrated that amino acid biosynthesis shifted in favor of nutrient transport and uptake in IBD. MGX studies investigating individuals with pediatric Crohn’s disease early on after diagnosis suggest that dysbiosis is the response of the microbial community towards inflammation, diet, and antibiotic exposure [111]. Longitudinal metagenomic studies also support the hypothesis of increased microbial oxidative stress in IBD [112], where these gene pathways were tightly correlated with a greater abundance of facultative anaerobe species and enrichment of *R. gnavus* in the gut of patients with IBD [112]. One of the aggregate conclusions of these studies is that the microbiota (as well as the host) is clearly under stress during dysbiosis in IBD, suggesting antioxidant therapies may hold potential to rescue both host inflammation and microbiota dysfunction. The effect of FMT on patients with Crohn’s disease was also interrogated with the use of shotgun MGX, and the findings suggested that an increased gut microbial diversity correlates with FMT efficacy, probably by restoring it closer to a healthy individual’s diversity [113]. A large Australian FMT study found that patients who achieved remission after FMT had increased levels of short-chain fatty acid biosynthesis and secondary bile acids compared with patients who did not [114]. Patients who did not achieve remission had increased levels of heme and lipopolysaccharide biosynthesis [114]. Again, these findings reflect restoration of a healthy gut metabolome state, but without further functional interrogation, this does not establish the cause or identify new molecular therapies.

Second Genome is a company that uses microbiome taxonomy research for fundamental therapeutic discovery and aims to innovate microbiome-based drugs. Their discovery platform uses a phage display technology to identify, screen, and validate microbe-derived peptides involved in the molecular interaction with the host. Selection of peptide candidates is based on analysis of datasets that comprise different types of omics data from healthy and IBD subsets. A protein currently under development for mucosal healing in this unique space is SG-2-0776 (Figure 1, Table 1).

Shotgun MGX has also been applied to stool samples from patients with IBD undergoing anti-integrin biologic therapy (vedolizumab) and revealed clear differences between patients that achieved remission versus patients refractory to treatment [110]. This study found that thirteen pathways were enriched at baseline in patients with CD who later went on to achieve remission at week 14, including the branched-chain amino acid (BCAA) biosynthesis pathways involved in the biosynthesis of L-citrulline, L-isoleucine from threonine, arginine, and polyamines [110]. For patients with UC, metabolite enrichment at baseline in patients that went on to achieve remission versus those that did not included increased lipid IVA biosynthesis, and decreased N10-formyl-tetrahydrofolate biosynthesis, pentose phosphate pathway, and pyruvate fermentation to lactate and acetate [110]. These pathways were significantly enriched in patients at baseline that achieved clinical remission following biologic treatment, suggesting these pathways may be predictive of response to therapy and/or may facilitate this clinical response together with vedolizumab [110]. Furthermore, at week 14 of follow-up, patients with UC that entered remission also showed substantial enrichment of gene pathways for pyruvate fermentation to lactate and acetate compared to patients that did not respond to treatment. Future studies with MGX followed up with functional interrogation in vitro and in vivo will need to identify greater specificity in bioactive metabolite pathways or microbial proteins that have potential to reverse biologic non-response in IBD.

While MGX sequencing relies on robust DNA integrity in samples to measure microbial gene abundance, it lacks the ability to distinguish between live and dead microbes as well as expressed and inactive/silent genes. The latter could be mistakenly highlighted in MGX study findings just because they are present in highly abundant species, although an inactive gene would bear no functional significance to disease. It is important to distinguish between these because the DNA abundance/‘potential’ of a microbiota gene is less important than the actual activity of a microbiota gene associated with disease.

### 6.3. Metatranscriptomics

In contrast to MGX, metatranscriptomics (MTX) quantifies the abundance of gene transcripts to assess gene activity, likely protein production, and therefore functionally significant genes (Table 2). Bacterial shotgun MTX, a variation of standard bulk RNA-seq, is particularly well suited to capture gene activity and is currently the only approach to look at transcriptional programs of microbiome genes and how they are altered in IBD [115,116]. MTX also has the powerful benefit that it can be paired in the same sample with MGX analysis to better capture both the microbial taxa present and their gene activity. Although MTX offers further power of microbial gene resolution and functional interrogation, there are only a few studies to date that have used MTX to investigate the microbiome. The disparity between numbers of MGX and MTX studies in IBD is due to the more recent advent of MTX technology and processing methods, as well as concerns over RNA stability and half-life. However, MTX studies are likely to see major growth in the future as they will be necessary to gain greater resolution at the functional level of interactions between the microbiota and disease.

The major issue with MTX analyses is the instability inherent to bacterial mRNA molecules; however, this is something that can be readily managed (Table 2) [117]. The relatively short half-life of mRNA molecules in combination with the high abundance of nucleases in stool and intestinal samples requires the use of methods that enable thorough preservation of RNA integrity [118]. Different methods have been trialed for sample collection by using untreated samples or adding different solvents to preserve fecal material such as ethanol or RNAlater, and these appear to achieve substantial improvements in mRNA preservation [77]. It can be argued that degradation of mRNA is not a uniform process, and that variation will result in some mRNA being degraded earlier than others. In addition to transcript-specific resistance to degradation, donor-specific retention times of the fecal material, for example, between patients with IBD and healthy controls, could create a bias in MTX analysis. Nevertheless, by following good practice for the design of all microbiome studies such as collection of large sample numbers, longitudinal sampling with multiple collections from each patient, and handling samples consistently and with an RNA preservative, the issues surrounding MTX can be overcome. In contrast to messenger RNA (mRNA), ribosomal RNA (rRNA) and transfer RNA (tRNA) are less prone to molecular degradation, and because of the natural tendency to be more stable, there is a bias towards rRNA. rRNA molecules outnumber mRNA and comprise over 80% of the total RNA [119]. Therefore, MTX samples contain large amounts of ribosomal RNA that need to either be eliminated before sequencing or filtered out during bioinformatics analysis [118,120,121]. MTX bioinformatic analysis follows much of the same process as MGX; however, it is improved by being able to map MTX to MGX reads aligned to reference genomes when studies analyze both DNA and RNA.

One of the first studies that explored the use of shotgun MTX on microbial communities was performed in combination with MGX and indicated that the relationship between gene copy number and transcripts varies across biological functions [122]. It is difficult to draw conclusions from this observation because it is possible that RNA degradation could be responsible, but it is just as possible that the DNA detected is actually a dead microbe, or an inactive or lowly expressed gene. An elegant study that evaluated different MTX protocols for sample handling was carried out by Franzosa et al. [77]. This group also investigated the relationship between MGX and MTX by correlating DNA abundance with its corresponding RNA expression. The authors found that ~41% of the microbial transcripts were not differentially expressed when compared to their genomic abundance [77]. These numbers suggest that the copy number of a gene is a major predictor of the expression of its corresponding transcript. Subsequent studies including larger numbers of paired fecal MGX and MTX samples showed inconsistencies among the species that were investigated between MGX and MTX, with Spearman’s correlations of 0.44 ± 0.10 (mean ± s.d.) [11]. These findings indicate that species’ abundance does not directly indicate their relative contribution to gene expression and implies that the current perspective on taxonomy and MGX sequencing needs to be further investigated.

A large multi-cohort multi-site longitudinal study of IBD subjects with paired MGX and MTX found that some species made very dominant contributions to the RNA transcriptional pool [11]. The genomic abundance of these species may have a deceptive effect, and considering their substantial transcriptional activity, the authors reasoned that loss of these species in IBD could therefore have major impacts in IBD [11], particularly *Faecalibacterium prausnitzii*, whose abundance has been previously linked to IBD [59]. Despite the relatively stable abundance of *F. prausnitzii* across IBD subjects, this species showed the highest degree of variation in functional activity (RNA), suggesting MGX was not predictive of its transcriptional activity [11]. In addition, the pathways with the lowest contributional diversity from taxa were dominated by *F. prausnitzii* [11]. Aside from dysbiosis occurring in the DNA abundance of taxa, it can also be observed in changes in RNA transcriptional activity within the microbiota [11]. This study identified that the most pronounced IBD-specific dysbiosis at the RNA level occurs within *R. gnavus*, which shows a significantly increased abundance of RNA transcripts in both UC and CD compared to non-IBD, despite the much smaller increases in DNA abundance [11]. Interestingly, several pathways such as the methylerythritol phosphate (MEP) pathway, which were expressed by microbes whose abundance correlated with IBD, showed enrichment only at the RNA level and not the DNA level [11]. By measuring transcriptional activity over multiple time points, the authors found that a shift in microbial species (i.e., from *Alistipes putredinis* to *B. vulgatus*) was the main contributor to the transcriptional activity changes of this MEP pathway, and this was associated with increased disease severity [11]. Another important example of MTX insight comes from highly abundant dead or inactive bacteria such as *Dialister invisus*, which is readily detected by MGX but shows little or no RNA expression. This highlights the importance of examining changes in MTX in multiple samples over time when exploring disease-specific transcriptional microbial signatures and observing shifts in the microbial pathway contribution.

From these studies, it can be concluded that MTX provides a valuable contribution to microbiome research as it captures functional differences in IBD that may be missed by MGX alone. However, MTX studies need to be accompanied by MGX technology to gain maximal insight into the role of the microbiome in disease.

Lloyd-Price et al. [12] concluded that a select subset of species including *Clostridium hathewayi*, *Clostridium bolteae*, and *R. gnavus* were significantly more abundant in IBD dysbiosis. In addition, the authors constructed a cross-measurement-type association network that included a range of microbiome measurements (MGX, MTX, and proteomics reflected as functional profiles such as Enzyme Commission (EC) gene families), metabolites, host intestinal biopsy transcription, and serology. This associated network confirmed earlier findings that *F. prausnitzii* accounted for some of the strongest associations in abundance and gene expression identified in dysbiosis, including the expression of numerous enzyme gene families that were downregulated in dysbiosis [12]. Finally, they found members of the *Roseburia* genus (together with *Subdoligranulum*) to be associated at both the DNA and RNA levels with metabolites dysregulated in IBD, such as acylcarnitines and bile acids [12].

Shotgun MTX studies comprise bulk RNA-seq technology that enables researchers to study all RNA molecules from all cells present in a mixed heterogenous sample, though it cannot distinguish transcripts at a single cell level. In contrast, single-cell RNA sequencing technology has the ability to study the transcriptome from a single cell. Although single-cell RNA-seq technology has made its way into mammalian cells, untargeted sequencing of RNA from single-cell prokaryotes has shown to be challenging due to the lower transcript numbers and the lack of a poly-A tail [123,124]. This technology will have great potential for future studies when used for high-throughput untargeted sequencing, since it will enable researchers to study phenotypic heterogeneity within genetically identical cells. It will also allow researchers to know precisely and with great accuracy what transcript is coming from which bacteria. This technology is in its infancy and has not yet been applied in high-throughput sequencing of complex microbial communities within the human gut. To date, its proof of concept has been used to sequence in vitro monocultures with split-pool indexing techniques on tens of thousands of bacterial cells [123,124]. Nevertheless, this is a rapidly evolving field, and the advent of commercially available kits for bacterial single-cell RNA-seq will likely have a positive impact on microbiome research.

## 7. Fractionation and Elimination Approach

Another method for microbiota-derived therapeutic candidate discovery is bioassay-guided fractionation. This approach has been used extensively in the discovery of plant-based natural products for several decades and is now being applied to human gut microbes. The process typically involves a crude extract first being isolated using different solvents, separating the material into fractions by their polarity [125]. All fractions are then assayed for bioactivity (e.g., using a cell line assay) followed by step-by-step fractionation of the most potent extract using various physical and chemical methods, such as size exclusion, high temperature or enzymatic treatment, in order to identify the broad chemical class of the compound. The detailed structure of the molecule is finally determined using chromatography and spectroscopy.

Based on the known changes in *R. gnavus* abundance in IBD linking it to disease, Henke et al. [73] used *R. gnavus* culture medium to stimulate murine bone marrow dendritic cells and demonstrated a dose-dependent increase in TNFα production. They then utilized size exclusion chromatography, followed by treatment with proteinases, nucleases, lysozyme, or boiling, which identified that the bioactive component was a polysaccharide. Finally, they employed chromatography and mass spectrometry to fully characterize the structure of the bioactive molecule to reveal it was a complex polysaccharide composed of a rhamnose backbone and glucose sidechains. This elegant functional interrogation of the bioactive components of one of the bacteria of importance in IBD suggests that this polysaccharide might contribute to higher disease activity and inflammation. Although it may be possible to design small molecule inhibitors to block this molecule, ultimately, the goal of these studies will be to identify therapeutic molecules to improve disease prognosis.

Another fractionation approach from Colosimo et al. [126] examined the interaction between gut microbial metabolites and G protein-coupled receptors (GPCRs). They screened the metabolites produced by seven common gut bacterial species (*Lactobacillus plantarum*, *Bifidobacterium longum*, *Faecalibacterium* *prauznitzii*, *Bacteroides vulgatus*, *Ruminococcus* *gnavus*, *Escherichia coli* *LF-82*, and *Enterococcus faecalis*), representing commensal, health-promoting, and pathogenic bacteria, against a bioassay of 241 GPCRs. A metabolite isolate from each species’ culture broth was partitioned using reversed-phase flash chromatography into nine fractions, which were tested separately for their ability to activate GPCRs. Bioassay-guided fractionation was used to then further purify specific metabolites from the bacterial culture broths. A strong agonist signal was reported between neurotransmitter GPCR receptors and aromatic amines produced by all microbiota species used in the study, suggesting these amines may be microbiota bioactive molecules important in intestinal neuronal signaling. Whether these aromatic amines are altered in IBD or could play a role in disease progression and hallmark symptoms of intestinal pain remains unclear. The major weakness with the fractionation-based approach is that it relies on using culturable strains of bacteria and culture broths, both of which underestimate the complexity of the gut microbiome and are unlikely to reproduce a substantial number of endogenous bioactive molecules.

## 8. Conclusions and Future Perspectives

Advances made in the last two decades of research have led to a clear understanding of the microbiome taxa changes in IBD, but we are only now reaching the point where we are beginning to unravel the functional gene units and bioactive molecules in the microbiota and how they may be responsible for regulating disease. There is enormous potential for therapeutic discoveries in this area. Future progress will necessitate a move away from FMT to more specific studies of individual and defined consortia of LBPs, metabolites, and critical microbial gene/protein therapeutic candidates. Discovery of these molecules is likely to involve a combination of sequencing-based approaches (MGX and MTX), metabolomics, and potentially microbe culture fractionation. Ongoing clinical trials of LBPs in IBD will yield important results for the field in the coming years and provide a guide as to the feasibility of the live microbe approach to treatment with regard to inter-individual variation and regulatory conditions. Discovery and development of microbiota-derived bioactive molecules is a relatively new field that is now expanding with much work remaining to be conducted, and in many cases, this means a time lag before candidates enter clinical trials.

## Figures and Tables

**Table 1:** Overview of microbiome-derived therapeutic candidates undergoing clinical trials.

Discovery	Company/Institute	Product Name	Mechanism	Product Type	NCT Number	Status
**Nucleic acid-based production**	Second Genome	SG-2-0776	Promotes mucosal healing	Protein	Not found	Proceeding to Phase 2
**Taxonomy based/probiotic**	Seres Therapeutics	Seres-287	Reduces gut inflammation	LBP	NCT03759041	Phase 2 active
	Vedanta	VE202	Treg induction	LBP	NCT03723746, NCT03931447	Phase 1 completed
	4D Pharma	Thetanix	Antagonizes NF-κB	LBP	NCT02704728	Phase 1 completed
	Enterome	EB-8018/Sibofimloc	FimH inhibition to block AIEC	Small molecule	NCT03709628, NCT03943446	Phase 2 recruiting
	Intralytix	EcoActive	Depletes AIEC	Bacteriophage	NCT03808103	Phase 1 recruiting
	BiomX	BX003 (BX002)	Targets *Klebsiella pneumoniae*	Bacteriophage cocktail	NCT04737876	Phase 1 completed
	Nordisk rebalance	Profermin	Reduces dysbiosis	Probiotic strains + fiber	NCT01245465, NCT01193894	Phase 2 and Phase 3 completed
**Metabolite based**	Brigham and Women’s Hospital	Butyrate with Hydroxocobalamin	Calprotectin reduction	Short-chain fatty acid	NCT04259060	Phase 2
	University of Padova	Micro-encapsulated sodium Butyrate	Treg and IL10 activation	Short-chain fatty acid	NCT04879914	Not applicable
	Stanford University	Ursodeoxycholic acid	Inflammatory marker reduction	Secondary bile acid	NCT03724175	Phase 2/3 recruiting
	University Medical Centre Groningen	Vitamin B2	Increase in the amount of *F. prausnitzii*	Vitamin metabolite	NCT02538354	Completed
	University Medical Centre Groningen	Vitamin B3	Reduction in inflammation and oxidative stress	Vitamin metabolite	NCT04913467	Phase 3

**Table 2:** Overview of strengths and limitations of the most common methods used in sequencing-based microbiome studies.

Sequencing Method	Strengths	Limitations
**16S rRNA**	Low cost for high throughput	Limited taxonomic resolution
	Lower complexity of bioinformatics analysis	Amplification bias
	Established taxonomic reference databases available	Does not capture gene content
**Shotgun Metagenomic Sequencing**	Sequences any organism present but only at high read depth	Higher cost
	Identifies functional potential and gene content	Higher complexity of bioinformatics analysis
		Host DNA contamination
**Shotgun Metatranscriptomic Sequencing**	Detects and quantifies microbial gene expression	Instability of mRNA
	Identifies active functional genes and pathways rather than DNA abundance of dead bacteria/silent genes	Host RNA contamination
	Identifies the relative importance and gene activity of similarly abundant microbial species	High abundance of ribosomal RNA

## References

[1] Lynch S.V., Pedersen O. (2016). The Human Intestinal Microbiome in Health and Disease. N. Engl. J. Med..

[2] Ungaro R., Mehandru S., Allen P.B., Peyrin-Biroulet L., Colombel J.F. (2017). Ulcerative colitis. Lancet.

[3] Torres J., Mehandru S., Colombel J.F., Peyrin-Biroulet L. (2017). Crohn’s disease. Lancet.

[4] Human Microbiome Project C. (2012). Structure, function and diversity of the healthy human microbiome. Nature.

[5] Manichanh C., Rigottier-Gois L., Bonnaud E., Gloux K., Pelletier E., Frangeul L., Nalin R., Jarrin C., Chardon P., Marteau P. (2006). Reduced diversity of faecal microbiota in Crohn’s disease revealed by a metagenomic approach. Gut.

[6] Ott S.J., Musfeldt M., Wenderoth D.F., Hampe J., Brant O., Folsch U.R., Timmis K.N., Schreiber S. (2004). Reduction in diversity of the colonic mucosa associated bacterial microflora in patients with active inflammatory bowel disease. Gut.

[7] Baumgart M., Dogan B., Rishniw M., Weitzman G., Bosworth B., Yantiss R., Orsi R.H., Wiedmann M., McDonough P., Kim S.G. (2007). Culture independent analysis of ileal mucosa reveals a selective increase in invasive *Escherichia coli* of novel phylogeny relative to depletion of Clostridiales in Crohn’s disease involving the ileum. ISME J..

[8] Frank D.N., St Amand A.L., Feldman R.A., Boedeker E.C., Harpaz N., Pace N.R. (2007). Molecular-phylogenetic characterization of microbial community imbalances in human inflammatory bowel diseases. Proc. Natl. Acad. Sci. USA.

[9] Morgan X.C., Tickle T.L., Sokol H., Gevers D., Devaney K.L., Ward D.V., Reyes J.A., Shah S.A., LeLeiko N., Snapper S.B. (2012). Dysfunction of the intestinal microbiome in inflammatory bowel disease and treatment. Genome Biol..

[10] Gevers D., Kugathasan S., Denson L.A., Vázquez-Baeza Y., Van Treuren W., Ren B., Schwager E., Knights D., Song S.J., Yassour M. (2014). The treatment-naive microbiome in new-onset Crohn’s disease. Cell Host Microbe.

[11] Schirmer M., Franzosa E.A., Lloyd-Price J., McIver L.J., Schwager R., Poon T.W., Ananthakrishnan A.N., Andrews E., Barron G., Lake K. (2018). Dynamics of metatranscription in the inflammatory bowel disease gut microbiome. Nat. Microbiol..

[12] Lloyd-Price J., Arze C., Ananthakrishnan A.N., Schirmer M., Avila-Pacheco J., Poon T.W., Andrews E., Ajami N.J., Bonham K.S., Brislawn C.J. (2019). Multi-omics of the gut microbial ecosystem in inflammatory bowel diseases. Nature.

[13] Darfeuille-Michaud A., Boudeau J., Bulois P., Neut C., Glasser A.-L., Barnich N., Bringer M.-A., Swidsinski A., Beaugerie L., Colombel J.-F. (2004). High prevalence of adherent-invasive *Escherichia coli* associated with ileal mucosa in Crohn’s disease. Gastroenterology.

[14] Reinisch W., Hébuterne X., Buisson A., Schreiber S., Desreumaux P., Paillarse J., Bonny C. (2020). P568 An open-label, multicenter, phase ib, pharmacokinetic (pk) and safety study of a fimh blocker, Sibofimloc (TAK-018/EB8018), in patients with Crohn’s disease (CD). J. Crohns Colitis.

[15] Youngster I., Russell G.H., Pindar C., Ziv-Baran T., Sauk J., Hohmann E.L. (2014). Oral, capsulized, frozen fecal microbiota transplantation for relapsing Clostridium difficile infection. JAMA.

[16] Bakken J.S., Borody T., Brandt L.J., Brill J.V., Demarco D.C., Franzos M.A., Kelly C., Khoruts A., Louie T., Martinelli L.P. (2011). Treating Clostridium difficile infection with fecal microbiota transplantation. Clin. Gastroenterol. Hepatol..

[17] DeFilipp Z., Bloom P.P., Torres Soto M., Mansour M.K., Sater M.R., Huntley M.H., Turbett S., Chung R.T., Chen Y.-B., Hohmann E.L. (2019). Drug-resistant *E. coli* bacteremia transmitted by fecal microbiota transplant. N. Engl. J. Med..

[18] Bennet J., Brinkman M. (1989). Treatment of ulcerative colitis by implantation of normal colonic flora. Lancet.

[19] Paramsothy S., Kamm M.A., Kaakoush N.O., Walsh A.J., van den Bogaerde J., Samuel D., Leong R.W., Connor S., Ng W., Paramsothy R. (2017). Multidonor intensive faecal microbiota transplantation for active ulcerative colitis: A randomised placebo-controlled trial. Lancet.

[20] Costello S.P., Hughes P.A., Waters O., Bryant R.V., Vincent A.D., Blatchford P., Katsikeros R., Makanyanga J., Campaniello M.A., Mavrangelos C. (2019). Effect of fecal microbiota transplantation on 8-week remission in patients with ulcerative colitis: A randomized clinical trial. JAMA.

[21] Rossen N.G., Fuentes S., van der Spek M.J., Tijssen J.G., Hartman J.H., Duflou A., Löwenberg M., van den Brink G.R., Mathus-Vliegen E.M., de Vos W.M. (2015). Findings from a randomized controlled trial of fecal transplantation for patients with ulcerative colitis. Gastroenterology.

[22] Moayyedi P., Surette M.G., Kim P.T., Libertucci J., Wolfe M., Onischi C., Armstrong D., Marshall J.K., Kassam Z., Reinisch W. (2015). Fecal microbiota transplantation induces remission in patients with active ulcerative colitis in a randomized controlled trial. Gastroenterology.

[23] Lewis J.D., Chuai S., Nessel L., Lichtenstein G.R., Aberra F.N., Ellenberg J.H. (2008). Use of the noninvasive components of the Mayo score to assess clinical response in ulcerative colitis. Inflamm. Bowel Dis..

[24] Imdad A., Nicholson M.R., Tanner-Smith E.E., Zackular J.P., Gomez-Duarte O.G., Beaulieu D.B., Acra S. (2018). Fecal transplantation for treatment of inflammatory bowel disease. Cochrane Database Syst Rev..

[25] Kaiko G.E., Stappenbeck T.S. (2014). Host–microbe interactions shaping the gastrointestinal environment. Trends Immunol..

[26] Donaldson G.P., Lee S.M., Mazmanian S.K. (2016). Gut biogeography of the bacterial microbiota. Nat. Rev. Microbiol..

[27] Ruff W.E., Greiling T.M., Kriegel M.A. (2020). Host–microbiota interactions in immune-mediated diseases. Nat. Rev. Microbiol..

[28] Guo C.-J., Allen B.M., Hiam K.J., Dodd D., Van Treuren W., Higginbottom S., Nagashima K., Fischer C.R., Sonnenburg J.L., Spitzer M.H. (2019). Depletion of microbiome-derived molecules in the host using Clostridium genetics. Science.

[29] Mazmanian S.K., Round J.L., Kasper D.L. (2008). A microbial symbiosis factor prevents intestinal inflammatory disease. Nature.

[30] Atarashi K., Tanoue T., Ando M., Kamada N., Nagano Y., Narushima S., Suda W., Imaoka A., Setoyama H., Nagamori T. (2015). Th17 cell induction by adhesion of microbes to intestinal epithelial cells. Cell.

[31] Viladomiu M., Kivolowitz C., Abdulhamid A., Dogan B., Victorio D., Castellanos J.G., Woo V., Teng F., Tran N.L., Sczesnak A. (2017). IgA-coated *E. coli* enriched in Crohn’s disease spondyloarthritis promote TH17-dependent inflammation. Sci. Transl. Med..

[32] Mishima Y., Sartor R.B. (2020). Manipulating resident microbiota to enhance regulatory immune function to treat inflammatory bowel diseases. J. Gastroenterol..

[33] Charbonneau M.R., Isabella V.M., Li N., Kurtz C.B. (2020). Developing a new class of engineered live bacterial therapeutics to treat human diseases. Nat. Commun..

[34] Ross J.J., Boucher P.E., Bhattacharyya S.P., Kopecko D.J., Sutkowski E.M., Rohan P.J., Chandler D.K., Vaillancourt J. (2008). Considerations in the development of live biotherapeutic products for clinical use. Curr. Issues Mol. Biol..

[35] Claesen J., Fischbach M.A. (2015). Synthetic microbes as drug delivery systems. ACS Synth. Biol..

[36] Schultz M. (2008). Clinical use of *E. coli* Nissle 1917 in inflammatory bowel disease. Inflamm. Bowel Dis..

[37] Dignass A., Lindsay J.O., Sturm A., Windsor A., Colombel J.-F., Allez M., D’Haens G., D’Hoore A., Mantzaris G., Novacek G. (2012). Second European evidence-based consensus on the diagnosis and management of ulcerative colitis part 2: Current management. J. Crohns Colitis.

[38] Losurdo G., Iannone A., Contaldo A., Ierardi E., Di Leo A., Principi M. (2015). *Escherichia coli* Nissle 1917 in ulcerative colitis treatment: Systematic review and meta-analysis. J. Gastrointestin. Liver Dis..

[39] Praveschotinunt P., Duraj-Thatte A.M., Gelfat I., Bahl F., Chou D.B., Joshi N.S. (2019). Engineered *E. coli* Nissle 1917 for the delivery of matrix-tethered therapeutic domains to the gut. Nat. Commun..

[40] Prantera C., Scribano M., Falasco G., Andreoli A., Luzi C. (2002). Ineffectiveness of probiotics in preventing recurrence after curative resection for Crohn’s disease: A randomised controlled trial with Lactobacillus GG. Gut.

[41] Cui H.-H., Chen C.-L., Wang J.-D., Yang Y.-J., Cun Y., Wu J.-B., Liu Y.-H., Dan H.-L., Jian Y.-T., Chen X.-Q. (2004). Effects of probiotic on intestinal mucosa of patients with ulcerative colitis. World J. Gastroenterol..

[42] Bjarnason I., Sission G. (2019). A randomised, double-blind, placebo-controlled trial of a multi-strain probiotic in patients with asymptomatic ulcerative colitis and Crohn’s disease. Inflammopharmacology.

[43] Sang L.-X., Chang B., Zhang W.-L., Wu X.-M., Li X.-H., Jiang M. (2010). Remission induction and maintenance effect of probiotics on ulcerative colitis: A meta-analysis. World J. Gastroenterol. WJG.

[44] Atarashi K., Tanoue T., Oshima K., Suda W., Nagano Y., Nishikawa H., Fukuda S., Saito T., Narushima S., Hase K. (2013). T reg induction by a rationally selected mixture of Clostridia strains from the human microbiota. Nature.

[45] Atarashi K., Tanoue T., Shima T., Imaoka A., Kuwahara T., Momose Y., Cheng G., Yamasaki S., Saito T., Ohba Y. (2011). Induction of colonic regulatory T cells by indigenous Clostridium species. Science.

[46] Misra B., Curran J., Herfarth H., Jagarlamudi K., Oneto C., Bhandari B., Wiener G., Kerman D., Moss A., Pomerantz R. (2018). P421 SER-287, an investigational microbiome therapeutic, induces remission and endoscopic improvement in a placebo-controlled, double-blind randomised trial in patients with active mild-to-moderate ulcerative colitis. J. Crohns Colitis.

[47] Wrzosek L., Miquel S., Noordine M.-L., Bouet S., Chevalier-Curt M.J., Robert V., Philippe C., Bridonneau C., Cherbuy C., Robbe-Masselot C. (2013). Bacteroides thetaiotaomicron and *Faecalibacterium prausnitzii* influence the production of mucus glycans and the development of goblet cells in the colonic epithelium of a gnotobiotic model rodent. BMC Biol..

[48] Dreher-Lesnick S.M., Stibitz S., Carlson J.J., Paul E. (2017). US regulatory considerations for development of live biotherapeutic products as drugs. Microbiol. Spectr..

[49] Cohen L.J., Cho J.H., Gevers D., Chu H. (2019). Genetic factors and the intestinal microbiome guide development of microbe-based therapies for inflammatory bowel diseases. Gastroenterology.

[50] Smillie C.S., Sauk J., Gevers D., Friedman J., Sung J., Youngster I., Hohmann E.L., Staley C., Khoruts A., Sadowsky M.J. (2018). Strain tracking reveals the determinants of bacterial engraftment in the human gut following fecal microbiota transplantation. Cell Host Microbe.

[51] Cieplak T., Soffer N., Sulakvelidze A., Nielsen D.S. (2018). A bacteriophage cocktail targeting *Escherichia coli* reduces *E. coli* in simulated gut conditions, while preserving a non-targeted representative commensal normal microbiota. Gut Microbes.

[52] Nakamoto N., Sasaki N., Aoki R., Miyamoto K., Suda W., Teratani T., Suzuki T., Koda Y., Chu P.-S., Taniki N. (2019). Gut pathobionts underlie intestinal barrier dysfunction and liver T helper 17 cell immune response in primary sclerosing cholangitis. Nat. Microbiol..

[53] Zhang A., Sun H., Wang P., Han Y., Wang X. (2012). Modern analytical techniques in metabolomics analysis. Analyst.

[54] Beale D.J., Pinu F.R., Kouremenos K.A., Poojary M.M., Narayana V.K., Boughton B.A., Kanojia K., Dayalan S., Jones O.A.H., Dias D.A. (2018). Review of recent developments in GC-MS approaches to metabolomics-based research. Metabolomics.

[55] Lavelle A., Sokol H. (2020). Gut microbiota-derived metabolites as key actors in inflammatory bowel disease. Nat. Rev. Gastroenterol. Hepatol..

[56] Smith P.M., Howitt M.R., Panikov N., Michaud M., Gallini C.A., Bohlooly Y.M., Glickman J.N., Garrett W.S. (2013). The microbial metabolites, short-chain fatty acids, regulate colonic Treg cell homeostasis. Science.

[57] Macia L., Tan J., Vieira A.T., Leach K., Stanley D., Luong S., Maruya M., Ian McKenzie C., Hijikata A., Wong C. (2015). Metabolite-sensing receptors GPR43 and GPR109A facilitate dietary fibre-induced gut homeostasis through regulation of the inflammasome. Nat. Commun..

[58] Parada Venegas D., De la Fuente M.K., Landskron G., Gonzalez M.J., Quera R., Dijkstra G., Harmsen H.J.M., Faber K.N., Hermoso M.A. (2019). Short Chain Fatty Acids (SCFAs)-Mediated Gut Epithelial and Immune Regulation and Its Relevance for Inflammatory Bowel Diseases. Front. Immunol..

[59] Machiels K., Joossens M., Sabino J., De Preter V., Arijs I., Eeckhaut V., Ballet V., Claes K., Van Immerseel F., Verbeke K. (2014). A decrease of the butyrate-producing species *Roseburia hominis* and *Faecalibacterium prausnitzii* defines dysbiosis in patients with ulcerative colitis. Gut.

[60] Huda-Faujan N., Abdulamir A.S., Fatimah A.B., Anas O.M., Shuhaimi M., Yazid A.M., Loong Y.Y. (2010). The impact of the level of the intestinal short chain Fatty acids in inflammatory bowel disease patients versus healthy subjects. Open Biochem. J..

[61] Scheppach W., Sommer H., Kirchner T., Paganelli G.M., Bartram P., Christl S., Richter F., Dusel G., Kasper H. (1992). Effect of butyrate enemas on the colonic mucosa in distal ulcerative colitis. Gastroenterology.

[62] Breuer R.I., Soergel K.H., Lashner B.A., Christ M.L., Hanauer S.B., Vanagunas A., Harig J.M., Keshavarzian A., Robinson M., Sellin J.H. (1997). Short chain fatty acid rectal irrigation for left-sided ulcerative colitis: A randomised, placebo controlled trial. Gut.

[63] Jamka M., Kokot M., Kaczmarek N., Bermagambetova S., Nowak J.K., Walkowiak J. (2021). The Effect of Sodium Butyrate Enemas Compared with Placebo on Disease Activity, Endoscopic Scores, and Histological and Inflammatory Parameters in Inflammatory Bowel Diseases: A Systematic Review of Randomised Controlled Trials. Complement. Med. Res..

[64] Jones B.V., Begley M., Hill C., Gahan C.G., Marchesi J.R. (2008). Functional and comparative metagenomic analysis of bile salt hydrolase activity in the human gut microbiome. Proc. Natl. Acad. Sci. USA.

[65] Ridlon J.M., Kang D.J., Hylemon P.B. (2006). Bile salt biotransformations by human intestinal bacteria. J. Lipid Res..

[66] Sinha S.R., Haileselassie Y., Nguyen L.P., Tropini C., Wang M., Becker L.S., Sim D., Jarr K., Spear E.T., Singh G. (2020). Dysbiosis-Induced Secondary Bile Acid Deficiency Promotes Intestinal Inflammation. Cell Host Microbe.

[67] Keely S.J., Steer C.J., Lajczak-McGinley N.K. (2019). Ursodeoxycholic acid: A promising therapeutic target for inflammatory bowel diseases?. Am. J. Physiol. Gastrointest. Liver Physiol..

[68] Kolho K.L., Pessia A., Jaakkola T., de Vos W.M., Velagapudi V. (2017). Faecal and Serum Metabolomics in Paediatric Inflammatory Bowel Disease. J. Crohns Colitis.

[69] Franzosa E.A., Sirota-Madi A., Avila-Pacheco J., Fornelos N., Haiser H.J., Reinker S., Vatanen T., Hall A.B., Mallick H., McIver L.J. (2019). Gut microbiome structure and metabolic activity in inflammatory bowel disease. Nat. Microbiol..

[70] Fornelos N., Franzosa E.A., Bishai J., Annand J.W., Oka A., Lloyd-Price J., Arthur T.D., Garner A., Avila-Pacheco J., Haiser H.J. (2020). Growth effects of N-acylethanolamines on gut bacteria reflect altered bacterial abundances in inflammatory bowel disease. Nat. Microbiol..

[71] Medema M.H., Kottmann R., Yilmaz P., Cummings M., Biggins J.B., Blin K., de Bruijn I., Chooi Y.H., Claesen J., Coates R.C. (2015). Minimum Information about a Biosynthetic Gene cluster. Nat. Chem. Biol..

[72] Ongey E.L., Giessmann R.T., Fons M., Rappsilber J., Adrian L., Neubauer P. (2018). Heterologous Biosynthesis, Modifications and Structural Characterization of Ruminococcin-A, a Lanthipeptide From the Gut Bacterium *Ruminococcus gnavus* E1, in *Escherichia coli*. Front. Microbiol..

[73] Henke M.T., Kenny D.J., Cassilly C.D., Vlamakis H., Xavier R.J., Clardy J. (2019). *Ruminococcus gnavus*, a member of the human gut microbiome associated with Crohn’s disease, produces an inflammatory polysaccharide. Proc. Natl. Acad. Sci. USA.

[74] Wlodarska M., Luo C., Kolde R., d’Hennezel E., Annand J.W., Heim C.E., Krastel P., Schmitt E.K., Omar A.S., Creasey E.A. (2017). Indoleacrylic Acid Produced by Commensal Peptostreptococcus Species Suppresses Inflammation. Cell Host Microbe.

[75] Quince C., Walker A.W., Simpson J.T., Loman N.J., Segata N. (2017). Shotgun metagenomics, from sampling to analysis. Nat. Biotechnol..

[76] Byrd D.A., Sinha R., Hoffman K.L., Chen J., Hua X., Shi J., Chia N., Petrosino J., Vogtmann E. (2020). Comparison of methods to collect fecal samples for microbiome studies using whole-genome shotgun metagenomic sequencing. mSphere.

[77] Franzosa E.A., Morgan X.C., Segata N., Waldron L., Reyes J., Earl A.M., Giannoukos G., Boylan M.R., Ciulla D., Gevers D. (2014). Relating the metatranscriptome and metagenome of the human gut. Proc. Natl. Acad. Sci. USA.

[78] Vaidya J.D., van den Bogert B., Edwards J.E., Boekhorst J., Van Gastelen S., Saccenti E., Plugge C.M., Smidt H. (2018). The effect of DNA extraction methods on observed microbial communities from fibrous and liquid rumen fractions of dairy cows. Front. Microbiol..

[79] Yuan S., Cohen D.B., Ravel J., Abdo Z., Forney L.J. (2012). Evaluation of methods for the extraction and purification of DNA from the human microbiome. PLoS ONE.

[80] Poretsky R., Rodriguez-R L.M., Luo C., Tsementzi D., Konstantinidis K.T. (2014). Strengths and limitations of 16S rRNA gene amplicon sequencing in revealing temporal microbial community dynamics. PLoS ONE.

[81] Young R.B., Marcelino V.R., Chonwerawong M., Gulliver E.L., Forster S.C. (2021). Key Technologies for Progressing Discovery of Microbiome-Based Medicines. Front. Microbiol..

[82] Ranjan R., Rani A., Metwally A., McGee H.S., Perkins D.L. (2016). Analysis of the microbiome: Advantages of whole genome shotgun versus 16S amplicon sequencing. Biochem. Biophys. Res. Commun..

[83] Amarasinghe S.L., Su S., Dong X., Zappia L., Ritchie M.E., Gouil Q. (2020). Opportunities and challenges in long-read sequencing data analysis. Genome Biol..

[84] Durazzi F., Sala C., Castellani G., Manfreda G., Remondini D., De Cesare A. (2021). Comparison between 16S rRNA and shotgun sequencing data for the taxonomic characterization of the gut microbiota. Sci. Rep..

[85] Douglas G.M., Maffei V.J., Zaneveld J.R., Yurgel S.N., Brown J.R., Taylor C.M., Huttenhower C., Langille M.G. (2020). PICRUSt2 for prediction of metagenome functions. Nat. Biotechnol..

[86] Callahan B.J., Wong J., Heiner C., Oh S., Theriot C.M., Gulati A.S., McGill S.K., Dougherty M.K. (2019). High-throughput amplicon sequencing of the full-length 16S rRNA gene with single-nucleotide resolution. Nucleic Acids Res..

[87] Johnson J.S., Spakowicz D.J., Hong B.-Y., Petersen L.M., Demkowicz P., Chen L., Leopold S.R., Hanson B.M., Agresta H.O., Gerstein M. (2019). Evaluation of 16S rRNA gene sequencing for species and strain-level microbiome analysis. Nat. Commun..

[88] Chehoud C., Albenberg L.G., Judge C., Hoffmann C., Grunberg S., Bittinger K., Baldassano R.N., Lewis J.D., Bushman F.D., Wu G.D. (2015). Fungal signature in the gut microbiota of pediatric patients with inflammatory bowel disease. Inflamm. Bowel Dis..

[89] Clooney A.G., Sutton T.D., Shkoporov A.N., Holohan R.K., Daly K.M., O’Regan O., Ryan F.J., Draper L.A., Plevy S.E., Ross R.P. (2019). Whole-virome analysis sheds light on viral dark matter in inflammatory bowel disease. Cell Host Microbe.

[90] Zuo T., Lu X.-J., Zhang Y., Cheung C.P., Lam S., Zhang F., Tang W., Ching J.Y., Zhao R., Chan P.K. (2019). Gut mucosal virome alterations in ulcerative colitis. Gut.

[91] Nelson M.T., Pope C.E., Marsh R.L., Wolter D.J., Weiss E.J., Hager K.R., Vo A.T., Brittnacher M.J., Radey M.C., Hayden H.S. (2019). Human and extracellular DNA depletion for metagenomic analysis of complex clinical infection samples yields optimized viable microbiome profiles. Cell Rep..

[92] Schmieder R., Edwards R. (2011). Fast identification and removal of sequence contamination from genomic and metagenomic datasets. PLoS ONE.

[93] Bharti R., Grimm D.G. (2021). Current challenges and best-practice protocols for microbiome analysis. Brief. Bioinform..

[94] Nurk S., Meleshko D., Korobeynikov A., Pevzner P.A. (2017). metaSPAdes: A new versatile metagenomic assembler. Genome Res..

[95] Boisvert S., Raymond F., Godzaridis É., Laviolette F., Corbeil J. (2012). Ray Meta: Scalable de novo metagenome assembly and profiling. Genome Biol..

[96] Peng Y., Leung H.C., Yiu S.-M., Chin F.Y. (2012). IDBA-UD: A de novo assembler for single-cell and metagenomic sequencing data with highly uneven depth. Bioinformatics.

[97] Luo R., Liu B., Xie Y., Li Z., Huang W., Yuan J., He G., Chen Y., Pan Q., Liu Y. (2012). SOAPdenovo2: An empirically improved memory-efficient short-read de novo assembler. Gigascience.

[98] Pell J., Hintze A., Canino-Koning R., Howe A., Tiedje J.M., Brown C.T. (2012). Scaling metagenome sequence assembly with probabilistic de Bruijn graphs. Proc. Natl. Acad. Sci. USA.

[99] Sedlar K., Kupkova K., Provaznik I. (2017). Bioinformatics strategies for taxonomy independent binning and visualization of sequences in shotgun metagenomics. Comput. Struct. Biotechnol. J..

[100] Jain M., Olsen H.E., Paten B., Akeson M. (2016). The Oxford Nanopore MinION: Delivery of nanopore sequencing to the genomics community. Genome Biol..

[101] Albertsen M., Hugenholtz P., Skarshewski A., Nielsen K.L., Tyson G.W., Nielsen P.H. (2013). Genome sequences of rare, uncultured bacteria obtained by differential coverage binning of multiple metagenomes. Nat. Biotechnol..

[102] Walker A.W., Duncan S.H., Louis P., Flint H.J. (2014). Phylogeny, culturing, and metagenomics of the human gut microbiota. Trends Microbiol..

[103] Almeida A., Mitchell A.L., Boland M., Forster S.C., Gloor G.B., Tarkowska A., Lawley T.D., Finn R.D. (2019). A new genomic blueprint of the human gut microbiota. Nature.

[104] Wood D.E., Salzberg S.L. (2014). Kraken: Ultrafast metagenomic sequence classification using exact alignments. Genome Biol..

[105] Truong D.T., Franzosa E.A., Tickle T.L., Scholz M., Weingart G., Pasolli E., Tett A., Huttenhower C., Segata N. (2015). MetaPhlAn2 for enhanced metagenomic taxonomic profiling. Nat. Methods.

[106] Kim D., Song L., Breitwieser F.P., Salzberg S.L. (2016). Centrifuge: Rapid and sensitive classification of metagenomic sequences. Genome Res..

[107] Tørresen O.K., Star B., Mier P., Andrade-Navarro M.A., Bateman A., Jarnot P., Gruca A., Grynberg M., Kajava A.V., Promponas V.J. (2019). Tandem repeats lead to sequence assembly errors and impose multi-level challenges for genome and protein databases. Nucleic Acids Res..

[108] Liao Y.-C., Lin S.-H., Lin H.-H. (2015). Completing bacterial genome assemblies: Strategy and performance comparisons. Sci. Rep..

[109] Woyke T., Doud D.F., Schulz F. (2017). The trajectory of microbial single-cell sequencing. Nat. Methods.

[110] Ananthakrishnan A.N., Luo C., Yajnik V., Khalili H., Garber J.J., Stevens B.W., Cleland T., Xavier R.J. (2017). Gut microbiome function predicts response to anti-integrin biologic therapy in inflammatory bowel diseases. Cell Host Microbe.

[111] Lewis J.D., Chen E.Z., Baldassano R.N., Otley A.R., Griffiths A.M., Lee D., Bittinger K., Bailey A., Friedman E.S., Hoffmann C. (2015). Inflammation, antibiotics, and diet as environmental stressors of the gut microbiome in pediatric Crohn’s disease. Cell Host Microbe.

[112] Hall A.B., Yassour M., Sauk J., Garner A., Jiang X., Arthur T., Lagoudas G.K., Vatanen T., Fornelos N., Wilson R. (2017). A novel *Ruminococcus gnavus* clade enriched in inflammatory bowel disease patients. Genome Med..

[113] Vaughn B.P., Vatanen T., Allegretti J.R., Bai A., Xavier R.J., Korzenik J., Gevers D., Ting A., Robson S.C., Moss A.C. (2016). Increased intestinal microbial diversity following fecal microbiota transplant for active Crohn’s disease. Inflamm. Bowel Dis..

[114] Paramsothy S., Nielsen S., Kamm M.A., Deshpande N.P., Faith J.J., Clemente J.C., Paramsothy R., Walsh A.J., van den Bogaerde J., Samuel D. (2019). Specific bacteria and metabolites associated with response to fecal microbiota transplantation in patients with ulcerative colitis. Gastroenterology.

[115] McNulty N.P., Yatsunenko T., Hsiao A., Faith J.J., Muegge B.D., Goodman A.L., Henrissat B., Oozeer R., Cools-Portier S., Gobert G. (2011). The impact of a consortium of fermented milk strains on the gut microbiome of gnotobiotic mice and monozygotic twins. Sci. Transl. Med..

[116] Maurice C.F., Haiser H.J., Turnbaugh P.J. (2013). Xenobiotics shape the physiology and gene expression of the active human gut microbiome. Cell.

[117] Kushner S.R. (2002). mRNA decay in *Escherichia coli* comes of age. J. Bacteriol..

[118] Deutscher M.P. (2006). Degradation of RNA in bacteria: Comparison of mRNA and stable RNA. Nucleic Acids Res..

[119] O’Neil D., Glowatz H., Schlumpberger M. (2013). Ribosomal RNA depletion for efficient use of RNA-seq capacity. Curr. Protoc. Mol. Biol..

[120] Giannoukos G., Ciulla D.M., Huang K., Haas B.J., Izard J., Levin J.Z., Livny J., Earl A.M., Gevers D., Ward D.V. (2012). Efficient and robust RNA-seq process for cultured bacteria and complex community transcriptomes. Genome Biol..

[121] He S., Wurtzel O., Singh K., Froula J.L., Yilmaz S., Tringe S.G., Wang Z., Chen F., Lindquist E.A., Sorek R. (2010). Validation of two ribosomal RNA removal methods for microbial metatranscriptomics. Nat. Methods.

[122] Turnbaugh P.J., Quince C., Faith J.J., McHardy A.C., Yatsunenko T., Niazi F., Affourtit J., Egholm M., Henrissat B., Knight R. (2010). Organismal, genetic, and transcriptional variation in the deeply sequenced gut microbiomes of identical twins. Proc. Natl. Acad. Sci. USA.

[123] Blattman S.B., Jiang W., Oikonomou P., Tavazoie S. (2020). Prokaryotic single-cell RNA sequencing by in situ combinatorial indexing. Nat. Microbiol..

[124] Kuchina A., Brettner L.M., Paleologu L., Roco C.M., Rosenberg A.B., Carignano A., Kibler R., Hirano M., DePaolo R.W., Seelig G. (2021). Microbial single-cell RNA sequencing by split-pool barcoding. Science.

[125] Bucar F., Wube A., Schmid M. (2013). Natural product isolation--how to get from biological material to pure compounds. Nat. Prod. Rep..

[126] Colosimo D.A., Kohn J.A., Luo P.M., Piscotta F.J., Han S.M., Pickard A.J., Rao A., Cross J.R., Cohen L.J., Brady S.F. (2019). Mapping Interactions of Microbial Metabolites with Human G-Protein-Coupled Receptors. Cell Host Microbe.

